# Perturbations in the neuroactive ligand-receptor interaction and renin angiotensin system pathways are associated with cancer-related cognitive impairment

**DOI:** 10.1007/s00520-025-09317-9

**Published:** 2025-03-06

**Authors:** Raymond J. Chan, Adam Walker, Janette Vardy, Alexandre Chan, Kate Oppegaard, Yvette P. Conley, Steven M. Paul, Kord M. Kober, Carolyn Harris, Joosun Shin, Lisa Morse, Ritu Roy, Adam Olshen, Marilyn J. Hammer, Jon D. Levine, Christine Miaskowski

**Affiliations:** 1 Flinders University, Bedford Park, Adelaide, South Australia Australia; 2 School of Clinical Medicine, University of New South Wales, Sydney, Australia; 3 Faculty of Medicine and Health, University of Sydney, Sydney, Australia; 4 School of Pharmacy and Pharmaceutical Sciences, University of California, Irvine, CA USA; 5 VA Portland Health Care System, Portland, OR USA; 6 School of Nursing, University of Pittsburgh, Pittsburgh, PA USA; 7 School of Nursing, University of California, San Francisco, CA USA; 8 Dana Farber Cancer Institute, Boston, MA USA; 9 School of Medicine, University of California, San Francisco, CA USA; 10 Department of Physiological Nursing, University of California, San Francisco, 490 Illinois Street, Floor 12, San Francisco, CA 94143 USA

**Keywords:** Cancer, Chemotherapy, Cognitive dysfunction, Cognitive impairment, Neuroinflammation, Renin angiotensin system

## Abstract

**Purpose:**

This study reports on the results from our data-driven approach that identified perturbations in neuroactive ligand-receptor interaction and renin-angiotensin system (RAS) pathways in oncology patients with and without self-reported cancer-related cognitive impairment (CRCI).

**Methods:**

In a sample of oncology patients receiving chemotherapy (*n* = 1343), the Attentional Function Index (AFI) was used to assess CRCI. Patients were grouped into low (AFI score of < 5) versus high (AFI score of > 7.5) levels of cognitive function. Gene expression analyses were done using RNA-seq (*n* = 185) and microarray (*n* = 158) technologies. Pathway impact analysis was used to evaluate for perturbations in biological pathways associated with self-reported CRCI.

**Results:**

The combined pathway impact analysis revealed that the neuroactive ligand-receptor interaction and RAS pathways were significantly perturbed between the patients with low versus high AFI scores.

**Conclusions:**

Findings from this study suggest that in addition to inflammatory pathways, numerous mechanisms may contribute to the underlying mechanisms for the development and/or persistence of self-reported CRCI.

**Supplementary Information:**

The online version contains supplementary material available at 10.1007/s00520-025-09317-9.

## Introduction

Cancer-related cognitive impairment (CRCI) occurs in 30% of patients prior to and in up to 75% of patients during chemotherapy [1]. For approximately 35% of patients, CRCI persists for months to years after treatment [1]. The clinical manifestations of CRCI include diminished short-term/working memory, as well as alterations in attention, executive function, and information processing speed [1]. This debilitating symptom is associated with significant decrements in work performance and productivity [2, 3], changes in family responsibilities [4], and decreases in all aspects of quality of life [2, 4].

Potential mechanisms for CRCI include blood brain barrier disruption, deoxyribonucleic acid (DNA) damage, telomere shortening, oxidative stress, neuroinflammation, and alterations in neurotransmission [5, 6]. As part of a data-driven investigation of the underlying mechanisms for CRCI, in our previous study [7], 12 Kyoto Encyclopedia of Genes and Genomes (KEGG) pathways were perturbed between oncology patients with high versus low levels of self-reported cognitive function. In the first publication from this analysis [7], perturbations in five inflammatory pathways (i.e., cytokine-cytokine receptor interaction, tumor necrosis factor signaling, mitogen-activated protein kinase signaling, interleukin-17 signaling, and mammalian target of rapamycin signaling) were described. These findings are supported by preclinical studies that demonstrated that anti-inflammatory interventions decrease cognitive symptoms associated with cancer or chemotherapy [8–10] and clinical studies that found positive associations between CRCI and pro-inflammatory cytokines [11–13].

As noted above, the mechanisms that underlie CRCI are complex and may interact to promote neuronal dysfunction and apoptosis and subsequent clinical manifestations of CRCI [5]. Evidence to support this hypothesis comes from studies of neurodegenerative disorders that identified that neuroinflammation results in alterations in neurotransmission [14–16]. For example, pro-inflammatory cytokines can increase the metabolism of dopamine in the hypothalamus, hippocampus, and nucleus accumbens [17]; lead to neurotoxicity by enhancing glutamatergic neurotransmission [18]; and increase astrocytic serotonin receptor transporter activity [19]. In addition, increases in pro-inflammatory cytokines induce a wide range of changes in multiple neurotransmitter systems (e.g., serotonin, dopamine, glutamate) [20]. Taken together, these findings support the hypothesis that alterations in various neurotransmitter systems contribute to the development and/or persistence of CRCI [5, 17].

It is interesting to note that the neuroactive ligand-receptor interaction pathway was one of the KEGG pathways identified in our initial data-driven analysis [7]. This signaling pathway is a collection of neurotransmitter receptors and ligands that play a role in a variety of physiological processes. Some of the neurotransmitters in this pathway include epinephrine, norepinephrine, dopamine, and serotonin. Perturbations in this pathway were identified in patients with Alzheimer’s disease (AD) [21] and in COVID-19 patients with dementia [22]. In addition, in a preclinical study, this pathway was associated with post-radiation cognitive dysfunction [23].

Another KEGG pathway identified in the initial analysis was the renin angiotensin system (RAS) pathway [7]. The actions of angiotensin II are mediated by angiotensin II type 1 (AT_1_) and angiotensin II type 2 (AT_2_) receptors, that have damage-inducing [24] and damage-protective [25] effects on neurons, respectively. Of note, alterations in RAS are being evaluated in patients with AD [26, 27]. Both pre-clinical [28–30] and clinical [31–33] studies have reported that the RAS in the brain is involved in the regulation of amyloid plaque deposition, neuroinflammation, oxidative stress, and vascular changes. All of these mechanisms are linked directly or indirectly with cognitive impairment in patients with AD [26]. While RASblockers were proposed as a potential preventative strategy for radiation-induced cognitive impairment [34], no studies have evaluated for associations between CRCI and the RAS in patients receiving chemotherapy. Therefore, this study extends our previous findings regarding associations between CRCI and inflammatory mechanisms [7] and reports on the results from our data-driven approach that identified perturbations in neuroactive ligand-receptor interaction and RAS pathways in oncology patients with and without self-reported CRCI.

## Methods

### Patients and settings

This study is part of a larger, longitudinal study of the symptom experience of oncology outpatients receiving chemotherapy whose details are published elsewhere [7]. Eligible patients were ≥ 18 years of age; had a diagnosis of one of the four most common cancers (i.e., breast, gastrointestinal, gynecological, or lung cancer); had received chemotherapy within the preceding four weeks; were scheduled to receive at least two additional cycles of chemotherapy; were able to read, write, and understand English; and gave written informed consent. Patients were recruited from two Comprehensive Cancer Centers, one Veteran’s Affairs hospital, and four community-based oncology programs in the United States.

### Study procedures

The study was approved by the Committee on Human Research at the University of California, San Francisco and by the Institutional Review Board at each of the study sites. Of the 2234 patients approached, 1343 consented to participate (60.1% response rate). The major reason for refusal was being overwhelmed with their cancer treatment. Eligible patients were approached in the infusion unit during their first or second cycle of chemotherapy by a member of the research team to discuss study participation and obtain written informed consent. The timing for the assessment of symptoms was selected because the prechemotherapy period is exceedingly stressful for patients. Data on self-reported cognitive function in the week prior to the patient’s second or third cycle of chemotherapy were used in this analysis. Blood for ribonucleic acid (RNA) isolation was collected at this time. Medical records were reviewed for disease and treatment information. For this study, a total of 717 patients provided a blood sample for the gene expression analyses (see Supplemental Fig. 1).

### Instruments

#### Demographic and clinical characteristics

Demographic information was obtained using a self-report questionnaire. Functional status was assessed using the Karnofsky Performance Status (KPS) scale [35]. The occurrence, treatment, and functional impact of 13 common medical conditions were assessed using the Self-Administered Comorbidity Questionnaire (SCQ) [36]. Alcohol consumption, behaviors, and associated problems were measured using the Alcohol Use Disorders Identification test (AUDIT) [37].

#### CRCI assessment

The 16-item Attentional Function Index (AFI) was used to assess patient’s self-reported level of cognitive function. The AFI assess an individual’s perceived effectiveness in performing daily activities that are supported by attention, working memory, and executive functions (i.e., setting goals, performing everyday tasks, and routine planning activities) [38]. A higher total mean score on a 0 to 10 numeric rating scale indicates better cognitive function. Total scores are grouped into categories of attentional function (i.e., < 5 low function, 5.0 to 7.5 moderate function, > 7.5 high function) [39]. Cronbach’s alpha for the total AFI score was 0.93.

### Acquisition and processing of gene expression data

The methods used for the gene expression analyses are described in detail elsewhere [40]. In brief, gene expression of total RNA isolated from peripheral blood of the 717 patients who provided a blood sample was quantified for 357 patients using RNA-sequencing (RNA-seq) and for 360 patients using microarray technologies. Two methods were used for the gene expression analyses because the availability of funds and the technologies themselves evolved over time.

### Data analyses

#### Demographic and clinical data

Demographic and clinical data from the two patient samples were analyzed separately using the IBM SPSS Statistics Version 27 (IBM Corporation, Armonk, NY). To evaluate for differences in gene expression using an extreme phenotype approach [41–43], patients who were classified into the two extreme groups based on their AFI scores (i.e., < 5 = low cognitive function versus > 7.5 = high cognitive function) were compared. For each gene expression platform, differences in demographic and clinical characteristics between the two groups were evaluated using parametric and non-parametric tests. Logistic regression analyses were used to determine significant covariates for inclusion in the differential expression analyses.

#### Differential expression and pathway impact analyses (PIA)

Differential expression was quantified using generalized linear models that were implemented separately for each sample (i.e., using edgeR [44] for RNA-seq and limma [45] for microarray). These analyses were adjusted for demographic and clinical characteristics that differed between patients who did and did not self-report CRCI. In addition, the models included surrogate variables not associated with CRCI to adjust for potential batch effects. Surrogate variable analysis is a statistical method that identifies and estimates sources of variation in high-throughput data. This method removes unwanted noise from sequencing data [46]. The differential expression results were summarized as the log fold-change and *p*-value for each gene. Only genes that had a common direction of expression (i.e., the same sign for the log fold-change) for both technologies were retained for subsequent analyses (*n* = 5235). Sequence loci data were annotated with Entrez gene identifiers. Gene symbols were annotated using the HUGO Gene Nomenclature Committee resource database [47]. The differential expression results of the two datasets were merged at the gene level using the Entrez gene identifiers. Fisher’s Combined Probability test was used to combine the differential gene expression results from both datasets using the uncorrected *p*-values [48].

To evaluate these results and interpret them in the context of CRCI-related mechanisms, PIAs were performed. This analysis includes potentially important biological factors (e.g., gene–gene interactions), the magnitude (i.e., log fold-change), and *p*-values from the combined differential expression analysis. This approach was chosen because PIA identifies biological pathways that are affected by a given perturbation (i.e., differences in the severity of cognitive impairment) and provides insights into potential underlying mechanisms [49].

Each PIA included the results of the combined differential expression analysis for all genes having a common direction of differential expression (i.e., cutoff free) to determine the probability of pathway perturbations (pPERT) using Pathway Express [50]. A total of 214 signaling pathways were defined using the KEGG database [51]. Fisher’s Combined Probability test was used to combine the PIA tests from both datasets using the uncorrected *p*-values [48]. The significance of the combined transcriptome-wide PIA analysis was assessed using a family wise error rate (FWER) of 1% under the Bonferroni method [50].

## Results

### RNA-Seq performance

As reported previously [7], of the 357 patients whose gene expression was quantified using RNA-seq, a total of 193 were in one of the extreme phenotype groups (i.e., AFI < 5 = Low group versus AFI > 7.5 = High group). Five of these patients were excluded as outliers or for poor RNA quantification. Of the remaining 188 evaluable patients, an additional three patients were excluded for missing phenotypic data. Of the remaining 185 patients whose phenotype data were evaluated, three patients were excluded from the gene expression analysis as outliers based on the multidimensional scaling plots. Median library threshold size was 9,273,000 reads. Following quality control filters, 13,301 genes were included in the final analysis. The common dispersion was estimated as 0.179, yielding a biological coefficient of variation of 0.423 well within the expected value for clinical samples [52]. Data from 185 patients were analyzed using RNA-seq.

### Microarray performance

As reported previously [7], of the 360 patients whose gene expression was quantified using microarray, a total of 179 were in one of the extreme phenotype groups (i.e., AFI < 5 = Low group versus AFI > 7.5 = High group). Three of these patients were excluded as outliers based on array intensity distributions evaluated using arrayQualityMetrics [53]. An additional 18 patients were excluded for missing phenotypic data. The phenotype data for the remaining 158 patients were evaluated. All of the samples demonstrated good hybridization performance based on biotin, background negative, and positive controls. Limma was used for background correction, quantile normalization, and log2 transformation [54]. Of the initial probes evaluated for quality (*n* = 46,542), 1953 probes had insufficient expression measurements (Illumina detection *p*-value < 0.05) and were excluded, leaving 44,589 probes for analysis.

### Logistic regression analyses

As reported previously [7], of the 185 evaluable patients in RNA-seq sample, 91 (49.2%) reported low AFI scores (Supplementary Table 1). In the logistic regression analysis for the RNA seq sample, seven phenotypic characteristics (i.e., age, ethnicity, current employment status, KPS score, SCQ score, self-reported diagnoses of depression, and cancer diagnosis) were retained in the final model.

Of the 158 evaluable patients in the microarray sample, 80 (50.6%) reported low AFI scores (Supplementary Table 2). In the logistic regression analysis for the microarray sample, four phenotypic characteristics (i.e., marital status, KPS score, self-reported diagnoses of depression and back pain) were retained in the final model (Supplementary Table 3).

### Differentially expressed genes and pathway impact analyses

As reported previously [7], of the 14 surrogate variables identified for RNA-seq sample, one was associated with AFI scores and was excluded from the final model. The final differential expression model for RNA-seq sample included 13 surrogate variables and the seven significant demographic and clinical characteristics. Of the 16 surrogate variables identified for the microarray sample, two were associated with AFI scores and were excluded from the final model. The final differential expression model for the microarray sample included 14 surrogate variables and the four significant demographic and clinical characteristics.

Fold changes and *p*-values for the differentially expressed genes were included in the PIAs of the 214 KEGG signaling pathways. Using Fisher’s combined probability method, in addition to the inflammatory pathways reported previously [7], the combined PIA analysis revealed that the neuroactive ligand-receptor interaction and RAS pathways were significantly perturbed between the AFI groups after correcting for multiple hypothesis testing using a common FWER of 1% (adjusted global perturbation *p*-value < 0.05) (Table 1).

**Table 1:** Perturbed KEGG pathways under investigation between patients with low and high Attentional Function Index scores

Pathway ID	Pathway name	Adjusted Global pPERT
hsa04614	Renin-angiotensin system	0.0067
hsa04080	Neuroactive ligand-receptor interaction	0.0009

*KEGG* Kyoto Encyclopedia of Genes and Genomes, *pPERT* perturbation *p*-value

## Discussion

This study is the first to describe associations between self-reported CRCI in patients receiving chemotherapy and perturbations in the neuroactive ligand-receptor interaction and RAS pathways. Based on the findings in our previous [7] and current study, it is reasonable to hypothesize that numerous mechanisms underlie the development and/or persistence of self-reported CRCI.

### Neuroactive ligand-receptor interaction pathway

The neuroactive ligand-receptor interaction pathway consists of over 300 genes associated with neuroreceptors and ligands that regulate a number of neurobiological functions including information processing through signaling molecules and receptor interactions. Changes in this pathway were found in studies of normal cognitive ageing [55, 56], AD [57, 58], and COVID-19 exacerbated dementia [22] as well as in studies of psychiatric or neurodevelopment disorders with impairments in cognitive function (e.g., schizophrenia [59], autism [60, 61]). Findings from the current study support previous research that suggests that potential underlying mechanisms for CRCI include alterations in neurotransmission and neuroreceptor signaling [5, 17].

Of note, in a study that used high-throughput transcriptional profiling with human prostate cancer cell culture models that mimicked androgen deprivation therapy (i.e., a treatment that is associated with cognitive impairment); biomarker selection using minimal common ontology elements-Cytoscape, and biomarker analyses using Advaita® iPathway Guide and DisGeNet for identification of disease-related genes [62], genes involved in multiple biological processes, including neuroactive receptor-ligand interaction and cytokine-cytokine receptor interaction (found in our previous study [7]) were identified. Functional enrichment and protein–protein interaction network analyses highlighted the role of ligand-gated ion channels and their receptors in cognitive dysfunction. The authors noted that these findings suggest potential biomarkers for the cognitive decline associated with androgen deprivation therapy in patients with prostate cancer. In another study, that performed differential expression and functional enrichment pathway analyses on hippocampal and lateral ventricle tissues from mice who did and did not receive cranial radiation [23], an association was found between radiation-induced cognitive dysfunction and the neuroactive receptor-ligand interaction pathway.

Although individual genes were not the focus of this study, given that inflammatory, neurotransmitter, and RAS genes are included in the neuroactive ligand-receptor interaction pathway and alterations in these systems are associated with CRCI [63], it is not surprising that perturbations in the neuroactive ligand-receptor interaction pathway were identified in the current study. A major challenge for future research will be to determine the critical components of this pathway that contribute to the development and/or persistence of CRCI. Refinements in animal models of CRCI (e.g., age, sex, genetic risk factors) are needed to determine the effects of perturbations in the neuroactive ligand-receptor interaction pathway in various brain regions as a result of different chemotherapy drugs [64, 65]. As is being done in AD [66], an evaluation of the features of T cells in peripheral blood and cerebrospinal fluid may be useful in exploring the neuroimmune-related mechanisms of CRCI. Equally important, given the large number of neurotransmitters involved in this pathway, research is needed on therapeutic interventions. Of note, preclinical [67] and network pharmacology [68] studies of traditional Chinese medicine formulations suggest that these treatments may alter the neuroactive ligand-receptor interaction pathway and warrant clinical evaluation.

### RAS pathway

While associations between cognitive changes in AD and alterations in the RAS are well documented [26, 27], no preclinical or clinical studies have evaluated for an association with self-reported CRCI. The RAS is a peptidergic system that plays a role in the regulation of cardiovascular and renal function. Specifically, it is involved in various processes including blood pressure regulation, neurotransmitter modulation, and neuroinflammation [69]. Within the brain, RAS plays a key role in the regulation of learning, memory, anxiety, depression, cognition and emotional stress. In addition, it is involved in the mechanisms that underlie stroke, dementia, and various neurodegenerative diseases [69].

In terms of memory and learning, AT_1_ receptor activation is associated with increases in oxidative stress [70] and cognitive impairment [71]. In contrast, AT_2_ receptor activation attenuates inflammation [72], oxidative stress [73], and abnormal neuronal firing [74]. Given that perturbations in inflammatory pathways were identified in our previous study of the same sample [7], it is reasonable to hypothesize that inflammation may play a role in changing the expression of these genes in a way that favors AT_1_ receptor signaling or diminishes AT_2_ expression in patients with CRCI. Preclinical and clinical studies are warranted to test this hypothesis.

Equally important, accumulating evidence suggests that RAS modulation or blockade is a potential therapeutic option for AD [75, 76]. For example, in a study that followed 2377 patients receiving angiotensin-converting enzyme inhibitors (ACEI) and 1780 patients receiving angiotensin II receptor blockers (ARB) for 12 years [77], all-cause dementia risks were reduced in individuals who received ACEIs and ARBs by 26% and 40%, respectively. In a systematic review of 15 randomized controlled trials that involved 34,666 elderly patients with hypertension [78], ARBs improved cognitive functioning particularly in terms of episodic memory. In addition, in a large retrospective epidemiological study that investigated the protective effects of prior use of ACEIs or ARBs on the risk for AD [27], only ARBs that crossed the blood brain barrier showed benefits. If the findings from the current study on RAS are replicated, future studies could evaluate the efficacy of these medications for the prevention and/or treatment of CRCI.

## Limitations

Several limitations warrant consideration. First, because subjective and objective measures of CRCI do not correlate [79–81], future studies should include both subjective and objective measures of CRCI. In addition, future studies should evaluate for associations between specific cognitive domains (e.g., memory, verbal fluency, executive functioning, multitasking [80]) and pathway perturbations. Because two technologies were used to perform the gene expression analyses, results may differ if all of the samples were run on the same platform. In addition, because our measures of CRCI and gene expression were done at a single time point (i.e., prior to the patients’ second or third cycle of chemotherapy), additional research is warranted to evaluate for changes in both subjective and objective evaluations of CRCI and pathway perturbations from prior to through the completion of chemotherapy.

## Conclusion

This study reports on associations between self-reported CRCI and perturbations in the neuroactive ligand-receptor interaction and RAS pathways. Additional research with preclinical models is needed to determine the causal role of these pathways in CRCI, as well as their potential interactions with inflammatory pathways. Transgenic mouse models may help identify the role of specific genes in the development and persistence of CRCI, as well as their influence within specific brain regions [82, 83]. Use of pre-clinical models of CRCI would allow for high-throughput drug screening to evaluate the efficacy of candidate pharmaceuticals that modulate the various neurotransmission pathways and RAS as potential agents to prevent or treat CRCI [84]. Additional clinical research is warranted to determine if subjective and objective measures of CRCI are associated with the same pathway perturbations.

## Supplementary Information

Below is the link to the electronic supplementary material.

Supplementary file1 (DOCX 32 KB)

Supplementary file2 (DOCX 29 KB)

Supplementary file3 (DOCX 30 KB)

Supplementary file4 (PDF 40 KB)

## Data Availability

Data are available from the corresponding author after the completion of a data sharing agreement with the University of California, San Francisco.
