# Supplementary material for: Perturbations in the neuroactive ligand-receptor interaction and renin angiotensin system pathways are associated with cancer-related cognitive impairment
Source: Support Care Cancer. 2025 Mar 6;33(4):254. doi: 10.1007/s00520-025-09317-9 (PMC11885406; doi:10.1007/s00520-025-09317-9)
Supplement: Supplementary file 1 — Supplementary file1 (DOCX 32 KB) [file 520_2025_9317_MOESM1_ESM.docx]

Supplementary Table 1. Differences in Demographic and Clinical Characteristics Between Patients in the RNA Seq Sample with High and Low Attentional Function Index Scores

| Characteristic | High AFI (score of >7.5)  50.8% (n=94) | Low AFI (score of <5)  49.2% (n=91) | Statistics |
| --- | --- | --- | --- |
|  | Mean (SD) | Mean (SD) |  |
| Age (years) | 58.4 (10.0) | 54.6 (13.2) | t = 2.23, p = 0.027 |
| Education (years) | 16.1 (3.1) | 15.7 (3.0) | t = 1.01, p = 0.315 |
| Body mass index (kg/m^2^) | 26.6 (5.1) | 27.0 (7.1) | t = -0.43, p = 0.665 |
| KPS score | 83.6 (11.6) | 72.0 (11.7) | t = 6.78, p < 0.001 |
| Number of comorbidities | 2.1 (1.2) | 3.1 (1.6) | t = -4.88, p < 0.001 |
| SCQ score | 4.5 (2.5) | 7.5 (4.0) | t = -6.13, p < 0.001 |
| AUDIT score | 2.7 (2.3) | 3.1 (3.1) | t = -0.90, p = 0.369 |
| Time since diagnosis (years) | 1.6 (2.9) | 1.9 (3.2) | U, p = 0.308 |
| Time since diagnosis (median) | 0.44 | 0.45 |  |
| Number of prior cancer treatments | 1.5 (1.3) | 1.6 (1.5) | t = -0.32, p = 0.751 |
| Number of metastatic sites including lymph node involvement | 1.3 (1.2) | 1.2 (1.2) | t = 0.65, p = 0.515 |
| Number of metastatic sites excluding lymph node involvement | 0.8 (1.0) | 0.7 (1.0) | t = 0.21, p = 0.837 |
| MAX2 score | 0.17 (0.08) | 0.19 (0.08) | t = -1.85, p = 0.066 |
|  | % (n) | % (n) |  |
| Gender  Female  Male | 68.1 (64)  31.9 (30) | 84.6 (77)  15.4 (14) | FE, p = 0.010 |
| Ethnicity  White  Black  Asian or Pacific Islander  Hispanic mixed or other | 64.9 (61)  13.8 (13)  9.6 (9)  11.7 (11) | 57.1 (52)  22.0 (20)  2.2 (2)  18.7 (17) | X^2^ = 7.90, p = 0.048  No significant post hoc contrasts |
| Married or partnered (% yes) | 63.7 (58) | 57.3 (51) | FE, p = 0.446 |
| Lives alone (% yes) | 19.6 (18) | 24.4 (22) | FE, p = 0.476 |
| Childcare responsibilities (% yes) | 17.8 (16) | 24.7 (22) | FE, p = 0.278 |
| Care of adult responsibilities (% yes) | 8.2 (7) | 11.0 (9) | FE, p = 0.606 |
| Currently employed (% yes) | 47.9 (45) | 24.2 (22) | FE, p = 0.001 |
| Income  <$30,000  $30,000 to <$70,000  $70,000 to <$100,000  ≥$100,000 | 18.4 (16)  19.5 (17)  24.1 (21)  37.9 (33) | 28.9 (24)  26.5 (22)  15.7 (13)  28.9 (24) | U, p = 0.044 |
| Specific comorbidities (% yes)  Heart disease  High blood pressure  Lung disease  Diabetes  Ulcer or stomach disease  Kidney disease  Liver disease  Anemia or blood disease  Depression  Osteoarthritis  Back pain  Rheumatoid arthritis | 3.2 (3)  29.8 (28)  4.3 (4)  11.7 (11)  6.4 (6)  0 (0)  5.3 (5)  6.4 (6)  6.4 (6)  10.6 (10)  21.3 (20)  2.1 (2) | 7.7 (7)  35.2 (32)  14.3 (13)  16.5 (15)  7.7 (7)  0 (0)  8.8 (8)  15.4 (14)  40.7 (37)  14.3 (13)  44.0 (40)  6.6 (6) | FE, p = 0.208  FE, p = 0.530  FE, p = 0.022  FE, p = 0.401  FE, p = 0.780  n/a  FE, p = 0.401  FE, p = 0.059  FE, p < 0.001  FE, p = 0.508  FE, p = 0.002  FE, p = 0.165 |
| Exercise on a regular basis (% yes) | 70.7 (65) | 62.1 (54) | FE, p = 0.268 |
| Smoking current or history of (% yes) | 26.1 (24) | 40.4 (36) | FE, p = 0.058 |
| Cancer diagnosis  Breast  Gastrointestinal  Gynecological  Lung | 34.0 (32)  45.7 (43)  14.9 (14)  5.3 (5) | 40.7 (37)  22.0 (20)  22.0 (20)  15.4 (14) | X^2^ = 14.04, p = 0.003  NS  0 > 1  NS  NS |
| Type of prior cancer treatment  No prior treatment  Only surgery, CTX, or RT  Surgery & CTX, or surgery & RT, or CTX & RT  Surgery & CTX & RT | 25.3 (23)  40.7 (37)  22.0 (20)  12.1 (11) | 25.8 (23)  44.9 (40)  14.6 (13)  14.6 (13) | X^2^ = 1.75, p = 0.627 |
| CTX cycle length  14 day cycle  21 day cycle  28 day cycle | 51.1 (48)  42.6 (40)  6.4 (6) | 37.4 (34)  56.0 (51)  6.6 (6) | X^2^ = 3.67, p = 0.159 |
| Emetogenicity of CTX  Minimal/low  Moderate  High | 17.0 (16)  69.1 (65)  13.8 (13) | 18.7 (17)  59.3 (54)  22.0 (20) | X^2^ = 2.48, p = 0.289 |
| Antiemetic regimens  None  Steroid alone or serotonin receptor antagonist alone  Serotonin receptor antagonist and steroid  NK-1 receptor antagonist and two other antiemetics | 4.3 (4)  17.4 (16)  54.3 (50)  23.9 (22) | 4.4 (4)  16.5 (15)  46.2 (42)  33.0 (30) | X^2^ = 1.95, p = 0.582 |
| AFI score at enrollment | 8.3 (0.7) | 3.8 (0.9) | t = 37.89, p < 0.001 |

Abbreviations: AFI = Attentional Function Index; AUDIT = Alcohol Use Disorders Identification Test; CTX = chemotherapy; FE = Fisher's exact test; kg = kilograms; KPS = Karnofsky Performance Status; m^2^ = meter squared, n/a = not applicable; NK-1 = neurokinin-1; NS = not significant; RNA = ribonucleic acid; RT = radiation therapy; SCQ = Self-administered Comorbidity Questionnaire; seq = sequencing; U = Mann-Whitney U test
