# Supplementary material for: Perturbations in the neuroactive ligand-receptor interaction and renin angiotensin system pathways are associated with cancer-related cognitive impairment
Source: Support Care Cancer. 2025 Mar 6;33(4):254. doi: 10.1007/s00520-025-09317-9 (PMC11885406; doi:10.1007/s00520-025-09317-9)
Supplement: Supplementary file 2 — Supplementary file2 (DOCX 29 KB) [file 520_2025_9317_MOESM2_ESM.docx]

Supplementary Table 2. Differences in Demographic and Clinical Characteristics Between Patients in the Microarray Sample with High and Low Attentional Function Index Scores

| Characteristic | High AFI (score of >7.5)  49.4% (n=78) | Low AFI (score of <5)  50.6% (n=80) | Statistics |
| --- | --- | --- | --- |
|  | Mean (SD) | Mean (SD) |  |
| Age (years) | 57.9 (10.5) | 53.6 (12.4) | t = 2.33, p = 0.021 |
| Education (years) | 16.6 (2.7) | 16.0 (3.0) | t = 1.44, p = 0.152 |
| Body mass index (kg/m^2^) | 25.6 (5.3) | 28.1 (6.2) | t = -2.69, p = 0.008 |
| KPS score | 83.8 (10.2) | 74.2 (10.9) | t = 5.72, p < 0.001 |
| Number of comorbidities | 2.1 (1.1) | 2.9 (1.5) | t = -3.33, p = 0.001 |
| SCQ score | 4.7 (2.4) | 6.6 (3.3) | t = -4.16, p < 0.001 |
| AUDIT score | 2.6 (1.8) | 3.2 (3.1) | t = -1.17, p = 0.247 |
| Time since diagnosis (years) | 2.5 (4.2) | 2.5 (3.6) | U, p = 0.143 |
| Time since diagnosis (median) | 0.42 | 0.61 |  |
| Number of prior cancer treatments | 1.9 (1.8) | 2.1 (1.6) | t = -0.86, p = 0.393 |
| Number of metastatic sites including lymph node involvement | 1.4 (1.4) | 1.2 (1.1) | t = 0.96, p = 0.336 |
| Number of metastatic sites excluding lymph node involvement | 0.9 (1.2) | 0.7 (1.0) | t = 0.98, p = 0.329 |
| MAX2 score | 0.17 (0.08) | 0.17 (0.08) | t = -0.26, p = 0.793 |
|  | % (n) | % (n) |  |
| Gender  Female  Male | 74.4 (58)  25.6 (20) | 85.0 (68)  15.0 (12) | FE, p = 0.115 |
| Ethnicity  White  Black  Asian or Pacific Islander  Hispanic, Mixed, or Other | 74.0 (57)  14.3 (11)  6.5 (5)  5.2 (4) | 67.5 (54)  11.3 (9)  11.3 (9)  10.0 (8) | X^2^ = 2.70, p = 0.440 |
| Married or partnered (% yes) | 82.1 (64) | 51.2 (41) | FE, p < 0.001 |
| Lives alone (% yes) | 11.5 (9) | 26.3 (21) | FE, p = 0.025 |
| Childcare responsibilities (% yes) | 20.8 (16) | 26.3 (21) | FE, p = 0.456 |
| Care of adult responsibilities (% yes) | 9.7 (7) | 12.2 (9) | FE, p = 0.792 |
| Currently employed (% yes) | 48.7 (38) | 21.3 (17) | FE, p < 0.001 |
| Income  <$30,000  $30,000 to <$70,000  $70,000 to <$100,000  ≥$100,000 | 10.3 (8)  12.8 (10)  14.1 (11)  62.8 (49) | 32.5 (26)  25.0 (20)  16.3 (13)  26.3 (21) | U, p < 0.001 |
| Specific comorbidities (% yes)  Heart disease  High blood pressure  Lung disease  Diabetes  Ulcer or stomach disease  Kidney disease  Liver disease  Anemia or blood disease  Depression  Osteoarthritis  Back pain  Rheumatoid arthritis | 6.4 (5)  24.4 (19)  14.1 (11)  6.4 (5)  2.6 (2)  1.3 (1)  7.7 (6)  14.1 (11)  7.7 (6)  11.5 (9)  16.7 (13)  1.3 (1) | 5.0 (4)  33.8 (27)  11.3 (9)  10.0 (8)  5.0 (4)  1.3 (1)  3.8 (3)  17.5 (14)  41.3 (33)  17.5 (14)  35.0 (28)  3.8 (3) | FE, p = 0.744  FE, p = 0.222  FE, p = 0.638  FE, p = 0.565  FE, p = 0.682  FE, p = 1.000  FE, p = 0.325  FE, p = 0.664  FE, p < 0.001  FE, p = 0.368  FE, p = 0.011  FE, p = 0.620 |
| Exercise on a regular basis (% yes) | 80.8 (63) | 63.7 (51) | FE, p = 0.021 |
| Smoking current or history of (% yes) | 32.1 (25) | 39.7 (31) | FE, p = 0.404 |
| Cancer diagnosis  Breast  Gastrointestinal  Gynecological  Lung | 37.2 (29)  20.5 (16)  24.4 (19)  17.9 (14) | 45.0 (36)  25.0 (20)  22.5 (18)  7.5 (6) | X^2^ = 4.40, p = 0.221 |
| Type of prior cancer treatment  No prior treatment  Only surgery, CTX, or RT  Surgery & CTX, or surgery & RT, or CTX & RT  Surgery & CTX & RT | 20.8 (16)  39.0 (30)  22.1 (17)  18.2 (14) | 10.0 (8)  47.5 (38)  22.5 (18)  20.0 (16) | X^2^ = 3.71, p = 0.294 |
| CTX cycle length  14 day cycle  21 day cycle  28 day cycle | 30.8 (24)  60.3 (47)  9.0 (7) | 41.3 (33)  52.5 (42)  6.3 (5) | X^2^ = 2.01, p = 0.366 |
| Emetogenicity of CTX  Minimal/low  Moderate  High | 21.8 (17)  59.0 (46)  19.2 (15) | 25.0 (20)  55.0 (44)  20.0 (16) | X^2^ = 0.30, p = 0.863 |
| Antiemetic regimens  None  Steroid alone or serotonin receptor antagonist alone  Serotonin receptor antagonist and steroid  NK-1 receptor antagonist and two other antiemetics | 12.2 (9)  20.3 (15)  50.0 (37)  17.6 (13) | 6.6 (5)  19.7 (15)  46.1 (35)  27.6 (21) | X^2^ = 3.06, p = 0.383 |
| Mean AFI score at enrollment | 8.4 (0.7) | 4.0 (0.8) | t = 36.03, p < 0.001 |

Abbreviations: AFI = Attentional Function Index; AUDIT = Alcohol Use Disorders Identification Test; CTX = chemotherapy; FE = Fisher's exact test; kg = kilograms; KPS = Karnofsky Performance Status; m^2^ = meter squared; NK-1 = neurokinin-1; RT = radiation therapy; SCQ = Self-administered Comorbidity Questionnaire; U = Mann-Whitney U test
