# Supplementary material for: Perturbations in the neuroactive ligand-receptor interaction and renin angiotensin system pathways are associated with cancer-related cognitive impairment
Source: Support Care Cancer. 2025 Mar 6;33(4):254. doi: 10.1007/s00520-025-09317-9 (PMC11885406; doi:10.1007/s00520-025-09317-9)
Supplement: Supplementary file 3 — Supplementary file3 (DOCX 30 KB) [file 520_2025_9317_MOESM3_ESM.docx]

Supplementary Table 3. Multiple Logistic Regression Analyses Predicting Low Attentional Function Index Group Membership

| RNA-seq Sample (n = 185) | | | |
| --- | --- | --- | --- |
| Predictors | Odds Ratio | 95% CI | p-value |
| Age | 0.96 | 0.92, 0.99 | 0.041 |
| Ethnicity  White  Black  Asian or Pacific Islander  Hispanic, Mixed, or Other | 1.00  0.10  2.31  1.44 | 0.01, 0.70  0.79, 6.69  0.45, 4.56 | 0.020  0.124  0.538 |
| Currently employed | 0.30 | 0.12, 0.74 | 0.009 |
| Karnofsky Performance Status scale score | 0.93 | 0.90, 0.97 | < 0.001 |
| Self-administered Comorbidity Questionnaire score | 1.24 | 1.06, 1.47 | 0.009 |
| Self-reported diagnosis of depression | 3.81 | 1.20, 12.17 | 0.024 |
| Cancer diagnosis  Breast cancer  Gastrointestinal cancer  Gynecological cancer  Lung cancer | 1.00  0.27  0.77  2.18 | 0.10, 0.72  0.24, 2.46  0.43, 10.99 | 0.008  0.654  0.343 |
| Overall model fit: df = 11, X^2^ = 101.00, p < 0.001 | | | |
| Microarray Sample (n = 158) | | | |
| Predictors | Odds Ratio | 95% CI | p-value |
| Married or partnered | 0.29 | 0.12, 0.69 | 0.005 |
| Karnofsky Performance Status scale score | 0.92 | 0.88, 0.96 | < 0.001 |
| Self-reported diagnosis of depression | 5.22 | 1.86, 14.68 | 0.002 |
| Self-reported diagnosis of back pain | 2.55 | 1.03, 6.33 | 0.043 |
| Overall model fit: df = 4, X^2^ = 61.49, p < 0.001 | | | |

Abbreviations: CI = confidence interval; df = degrees of freedom; RNA = ribonucleic acid; seq = sequencing
