# Supplementary material for: Perturbations in the neuroactive ligand-receptor interaction and renin angiotensin system pathways are associated with cancer-related cognitive impairment
Source: Support Care Cancer. 2025 Mar 6;33(4):254. doi: 10.1007/s00520-025-09317-9 (PMC11885406; doi:10.1007/s00520-025-09317-9)
Supplement: Supplementary file 4 — Supplementary file4 (PDF 40 KB) [file 520_2025_9317_MOESM4_ESM.pdf]

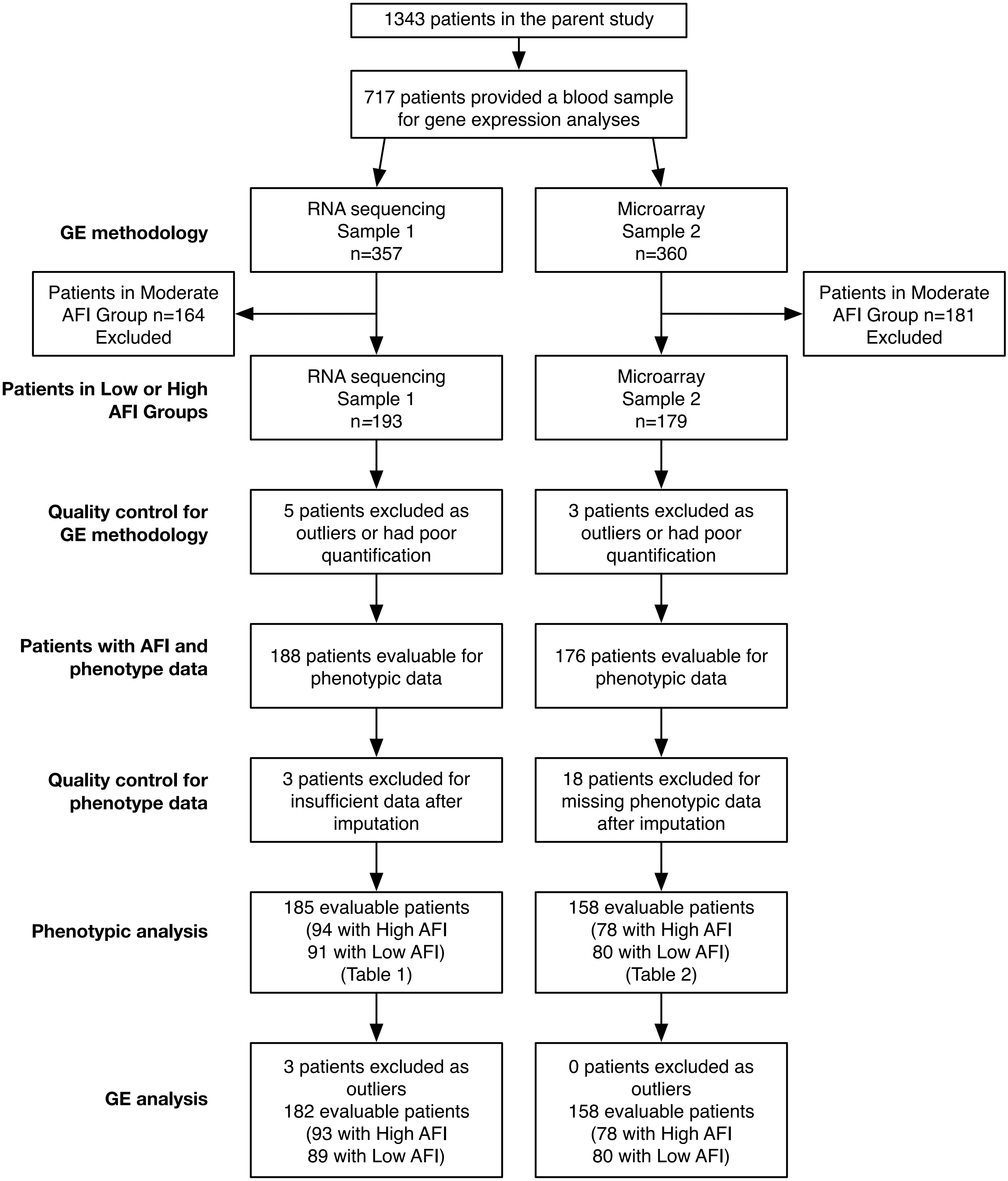

Supplementary Figure 1: Flow diagram of number of patients available for phenotypic and gene expression (GE) analyses for the cancer-related cognitive impairment (CRCI) extreme phenotype analysis. CRCI was assessed using the Attentional Function Index (AFI).
